# Acute maternal oxidant exposure causes susceptibility of the fetal brain to inflammation and oxidative stress

**DOI:** 10.1186/s12974-017-0965-8

**Published:** 2017-09-30

**Authors:** Feroz Akhtar, Christopher A. Rouse, Gabriel Catano, Marcus Montalvo, Sarah L. Ullevig, Reto Asmis, Kusum Kharbanda, Shivani K. Maffi

**Affiliations:** 1 School of Medicine, Department of Biomedical Sciences, Regional Academic Health Center, University of Texas Rio Grande Valley, 1204 W. Schunior, Edinburg, 78241 TX USA; 2 Department of Pediatrics, Uniformed Services University of Health Sciences & Walter Reed National Military Medical Center, Jones Bridge Rd, Bethesda, MD USA; 3 Department of Medicine, The University of Texas Health Science Center, San Antonio, TX USA; 4 Department of Kinesiology, Health, and Nutrition, University of Texas at San Antonio, San Antonio, TX USA; 5 Department of Clinical Lab Sciences, The University of Texas Health Science Center, San Antonio, TX USA; 6 Department of Internal Medicine, University of Nebraska Health Science Center, Omaha, NE USA

**Keywords:** Glutathione, Inflammation, Microglia, Oxidative stress, NF-kB, Fetal alcohol syndrome

## Abstract

**Background:**

Maternal exposure to environmental stressors poses a risk to fetal development. Oxidative stress (OS), microglia activation, and inflammation are three tightly linked mechanisms that emerge as a causal factor of neurodevelopmental anomalies associated with prenatal ethanol exposure. Antioxidants such as glutathione (GSH) and CuZnSOD are perturbed, and their manipulation provides evidence for neuroprotection. However, the cellular and molecular effects of GSH alteration in utero on fetal microglia activation and inflammation remain elusive.

**Methods:**

Ethanol (EtOH) (2.5 g/kg) was administered to pregnant mice at gestational days 16–17. One hour prior to ethanol treatment, *N*-acetylcysteine (NAC) and L-buthionine sulfoximine (BSO) were administered to modulate glutathione (GSH) content in fetal and maternal brain. Twenty-four hours following ethanol exposure, GSH content and OS in brain tissues were analyzed. Cytokines and chemokines were selected based on their association with distinctive microglia phenotype M1-like (IL-1β, IFN γ, IL-6, CCL3, CCL4, CCL-7, CCL9,) or M2-like (TGF-β, IL-4, IL-10, CCL2, CCL22, CXCL10, Arg1, Chi1, CCR2 and CXCR2) and measured in the brain by qRT-PCR and ELISA. In addition, Western blot and confocal microscopy techniques in conjunction with EOC13.31 cells exposed to similar ethanol-induced oxidative stress and redox conditions were used to determine the underlying mechanism of microglia activation associated with the observed phenotypic changes.

**Results:**

We show that a single episode of mild to moderate OS in the last trimester of gestation causes GSH depletion, increased protein and lipid peroxidation and inflammatory responses inclined towards a M1-like microglial phenotype (IL-1β, IFN-γ) in fetal brain tissue observed at 6–24 h post exposure. Maternal brain is resistant to many of these marked changes. Using EOC 13.31 cells, we show that GSH homeostasis in microglia is crucial to restore its anti-inflammatory state and modulate inflammation. Microglia under oxidative stress maintain a predominantly M1 activation state. Additionally, GSH depletion prevents the appearance of the M2-like phenotype, while enhancing morphological changes associated with a M1-like phenotype. This observation is also validated by an increased expression of inflammatory signatures (IL-1β, IFN-γ, IL-6, CCL9, CXCR2). In contrast, conserving intracellular GSH concentrations eliminates OS which precludes the nuclear translocation and more importantly the phosphorylation of the NFkB p105 subunit. These cells show significantly more pronounced elongations, ramifications, and the enhanced expression of M2-like microglial phenotype markers (IL-10, IL-4, TGF-β, CXCL10, CCL22, Chi, Arg, and CCR2).

**Conclusions:**

Taken together, our data show that maintaining GSH homeostasis is not only important for quenching OS in the developing fetal brain, but equally critical to enhance M2 like microglia phenotype, thus suppressing inflammatory responses elicited by environmental stressors.

**Electronic supplementary material:**

The online version of this article (10.1186/s12974-017-0965-8) contains supplementary material, which is available to authorized users.

## Background

Clinical and preclinical evidence indicates that maternal oxidative stress [1–3] and immune activation are a major source of non-genomic alterations that impair fetal neurodevelopment [4, 5]. Anomalies arise because fetal brain growth and plasticity is strongly influenced by the local cellular metabolic milieu and is highly susceptible to neurochemical perturbations caused by prenatal factors, such as inflammation and environmental stressors [6–8]. Ethanol is a common prenatal environmental stressor known to cause neurocognitive deficits and behavioral abnormalities, the broad effects of which are clinically categorized as Fetal Alcohol Spectrum Disorder (FASD) [2]**.** Irreversible fetal brain damage occurs due to the loss of glia and neurons in various brain compartments [9–11], the degree of injury being directly dependent on dose, duration of ethanol exposure, and gestational age of the unborn fetus [9, 12]. Additionally, neurons are particularly sensitive to the effects of ethanol during the period of synaptogenesis, also known as the brain growth spurt period, generally observed in the second trimester in rodents and during the last trimester of gestation in humans [9]. The molecular mechanisms underlying FASD are yet to be fully understood; however, there is evidence that increased oxidative stress [13], diminished antioxidant enzymes [1, 14], and more recent reports suggest neuro-immune activation and inflammation [11, 12, 15, 16] all contribute significantly towards ethanol-induced neurotoxicity.

Microglia are the resident immune cells of the central nervous system (CNS). Activation and polarization of microglia is regulated by both endogenous and exogenous factors [17]. Animal models of adult ethanol consumption further indicate that the damaging effects on the brain are due to priming and activation of microglia [18–20], leading to sustained inflammation which is in turn driven by a surge of pro-inflammatory cytokines, such as TNF-α, IL-1β, and IL-6 [21–24]. Depending on molecular signals received by the microglia receptors, activated microglia acquire either a “cytotoxic M1” or an “alternatively activated M2” neuroprotective phenotype [25]. M1-like phenotype is associated with reduced neurogenesis and deterioration of the neurotrophic system due to the release of pro-inflammatory mediators such as IFN-γ, IL-1β, IL-6, and TNF-α [26, 27]. On the other hand, a M2-like phenotype triggers an array of neuroprotective chemokines. Moreover, microglia’s conversion into either classical or alternative phenotype appears to be controlled by two transcriptional factors: nuclear factor kappa-light-chain-enhancer of activated B cells (NFκB) and nuclear factor (erythroid-derived2)-like 2 (Nrf-2), both extremely sensitive to oxidative stress and redox signaling [28]. While ROS signaling molecules augment p65/p50 dimer formation that subsequently leads to NFκB-dependent transcription of inflammatory cytokines/chemokines associated with a M1-like phenotype, Nrf-2 protects against oxidative damage [29] by activating genes involved in the synthesis of antioxidant enzymes and may likely be a critical regulator of the M2-like phenotype [28]. Despite this knowledge, the synergy of these two transcription factors in modulating microglia function during fetal development and under redox dysfunction remains poorly understood.

In utero ethanol exposure directly activates microglia through TLR2 and TLR4, triggering both oxidative stress and inflammatory cytokine production [11]. Therefore, intracellular factors such as antioxidants (GSH) are likely to influence microglia function [28]. However, it is unclear how and to what extent changes in maternal redox homeostasis impact gestational immune environment and its ultimate influence on microglia activation in the developing fetal brain. We hypothesized that even a single episode of endogenous glutathione dysregulation in utero around mid-gestation is sufficient to cause oxidative stress and inflammatory imprint in the fetal brain. Therefore, in this study, we utilized an in vivo and in vitro *model* of a short-term shift in oxidative-redox balance to determine the corresponding effect on fetal immune response associated with microglia M1/M2 phenotypic shift.

## Methods

### In vivo experimental protocol

#### Animal model

Timed pregnant C57 B6 mice were obtained directly from Harlan Laboratories (Indianapolis, IN) at or around gestational day 11. These mice were housed and acclimatized in the 59th Clinical Research Division (San Antonio, TX) animal facility until the day of the experiment. All parameters mentioned below were evaluated in fetal mice of either sex obtained at gestational days 17–18. Seven dams (maternal mice) were placed in each of the following treatment groups: control, EtOH only, NAC only, NAC/EtOH, BSO, and BSO/EtOH totaling 42 total dams.

#### Treatment

**Fig. 1:**
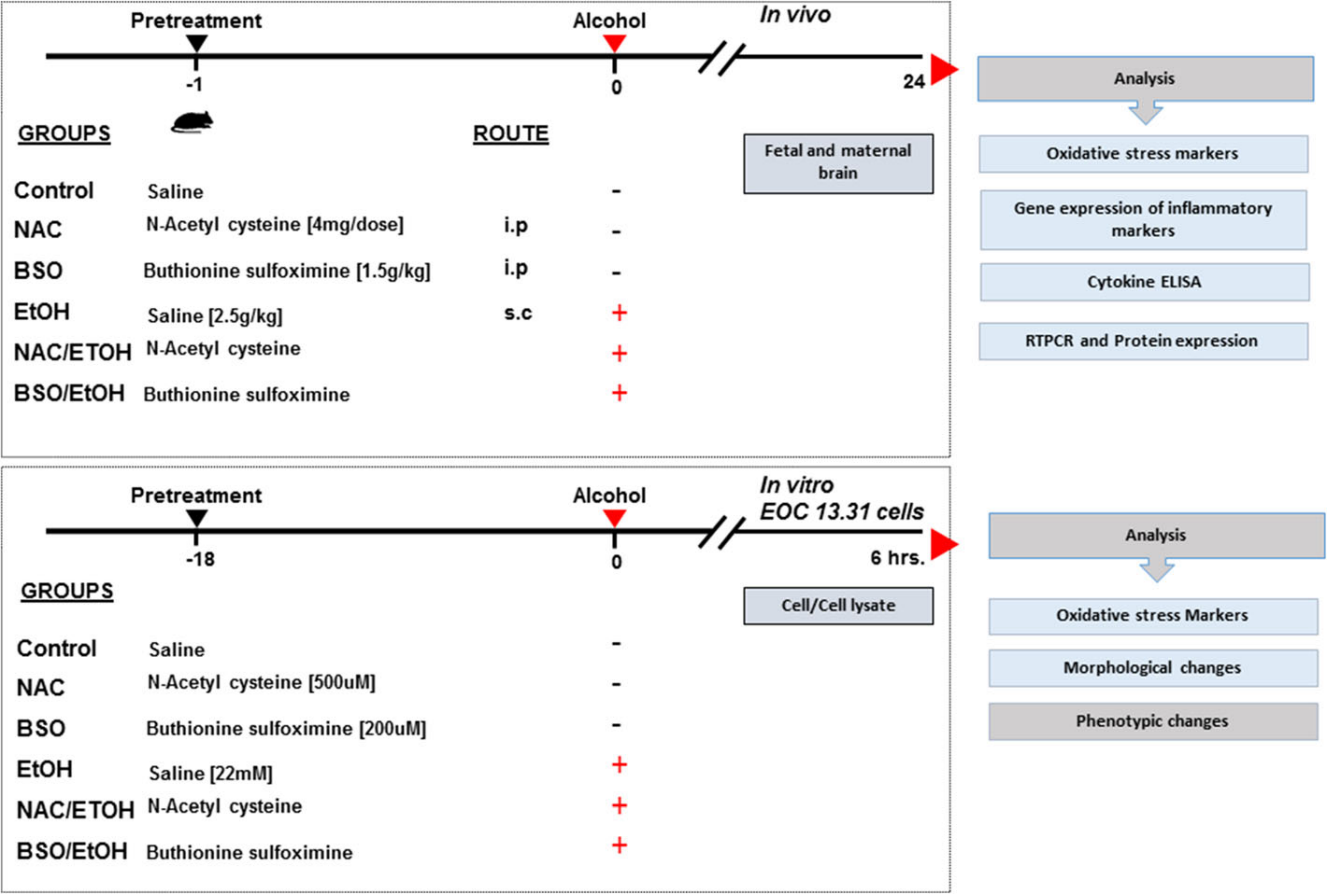
Overview of the experimental design. Top panel in in vivo studies, pregnant C57 B6 mice were divided into the following groups: Control, NAC, BSO, EtOH, EtOH/NAC, and BSO/EtOH. For combined treatments, NAC or BSO was administered 1 h prior to ethanol exposure. Maternal and fetal brain samples were used for further experiments. Bottom panel In vitro model using EOC13.31 cells (microglia cell line) were classified similar to the above 6 groups. Microglia was exposed with NAC or BSO for 18 h followed by 6-h ethanol treatment. Live cells and cell lysates were processed further for analysis of oxidative stress, inflammatory, and morphological changes

Ethanol, diluted in 20% saline, was administered to dams subcutaneously at a dose of 2.5 g/kg. BSO, 1.5 g/kg, and NAC, 4 mg/dose, intraperitoneal treatments were administered 1 h prior to alcohol treatment. Twenty-four hours following ethanol exposure, the maternal dams were humanely euthanized via cervical dislocation and the *fetuses* were harvested. For RNA and protein expression experiments, maternal and fetal brain tissues were flash frozen in liquid nitrogen and stored at − 80 °C until used. A description of the treatment regimen, dosing, and route of administration is provided in Fig. 1.

### In vitro microglia culture protocol

#### Cell culture

Murine microglia cell line (EOC13.31) was obtained from the American Type Culture Collection (ATCC; Manassas, VA, USA). Cells were maintained in Dulbecco’s modified Eagle’s medium with 4 mM L-glutamine adjusted to contain 1.5 g/l sodium bicarbonate and 4.5 g/l glucose, 70%; fetal bovine serum, 10%; and LADMAC conditioned media (produced from the LADMAC cell line (CRL-2420), 20%, and kept at 37 °C in a 5% CO2 incubator. The culture medium was regularly replenished at 3–4-day interval.

#### Cell treatment

Microglia were seeded at the density of 2 × 10^5^ cells/mL in either 60 or 100 mm dishes and were pretreated overnight (~ 18–20 h) with PBS (control), NAC (500 μM), and BSO (200 μM) diluted in PBS [10]. One set from each treated group was further exposed to 22 mM (~ 1 mg/ml) ethanol for 6 h. A small ethanol-filled beaker was placed in the incubator to maintain optimum ethanol concentration in the culture media at all times [10].

#### Total glutathione levels in brain lysates

Brain extracts were subjected to HPLC analysis for the determination of GSH levels as detailed in [30] and [31] with slight modification. Briefly, brain lysates were prepared in Tris-EDTA buffer + protease inhibitor cocktail (P8340, Sigma Aldrich) to yield a concentration of 30-μg protein/Eppendorf tube. To alkylate the free thiol groups, 100 mM of freshly prepared *N*-ethylmaleimide (NEM) was added to the samples. Proteins were precipitated with 40 μl cold 18% perchloric acid (PCA). One hundred fifty-microliter aliquots of the supernatant obtained were neutralized with 2 M KPi and reduced with dithiothreitol (DTT) (final concentration 6.9 mM). Each sample was diluted with 500 μl of 0.1 M KPi followed by o-phthalaldehyde (final concentration 11.2 mM, DTT < 2.4 mM) to obtain glutathione derivatives, which were subsequently separated by reverse phase HPLC. HPLC analysis was performed on a Jasco HPLC system equipped with a spectrofluorometer (FP-920, Jasco Inc.) set to an excitation wavelength of 340 nm and an emission wavelength of 420 nm. Glutathione was separated isocratically on a Brownlee 3-cm C18 ODS guard column (5 μm) and a Brownlee 22-cm C18 ODS analytical column (5 μm) with 21 mM propionate buffer (in 35 mM NaPi, pH 6.5)/acetonitrile (95/5 by volume) at a flow rate of 1.2 ml/min.

#### Protein carbonyl content

The reactive carbonyl contents in brain lysates were measured by the widely applied 2, 4-dinitrophenylhydrazine (DNPH) procedure [32]. Proteins (1–1.5 mg/ml) were separated into two 200-μl aliquots (i.e., a test sample and a blank sample). One milliliter of 10 mM DNPH in 2.5 M HCl was added to the test sample fraction while 1.0 ml of 2.5 M HCl alone was added to the blank sample fraction. Both fractions were then incubated in the dark at room temperature for 15 min. The samples were precipitated with 1.0 ml of 20% trichloroacetic (TCA) on ice for 5 min followed by centrifugation at 10,000*g* for 10 min. Subsequently, the tubes were treated with a 10% TCA wash, followed by washing in ethanol:ethyl acetate (1:1, *v*/*v*, four times). The final pellets were dissolved in 500 μl of 6 M guanidine-HCl in the presence of 20 mM phosphate buffer:trifluoroacetic acid (pH 2.3) and left vortexing for 30 min at 50 °C. The reactive carbonyl content was calculated from its peak absorption at 370 nm using a molar absorption coefficient (ε) of 22,000 M^−1^ cm^−1^. Reactive carbonyl content (μmol/l) was calculated using Beer-Lambert equation: Abs380nm (test-blank) × 10^6^/ε. The final carbonyl content in the protein was expressed as μmol/mg protein.

#### Reverse transcriptase polymerase chain reaction (RT-PCR)

Total RNA was extracted from fetal brains or microglia cells using RNeasy mini kit (Qiagen, Valencia, CA) following the manufacturer’s instructions (Catalog 74104). Quantification and analysis of nucleic acid purity were performed with spectrophotometry (NanoDrop Technologies, Wilmington, DE), and 1 μg of each sample was reverse transcribed with Moloney murine leukemia virus reverse transcriptase (Superscript II First-Strand Synthesis System for RT-PCR, Invitrogen) in a 20 μl of reaction mixture using oligo (dT) primer. Gene expression was measured using real-time PCR. The following primers and FAM-labeled probes from Applied Biosystems Inventoried Assays were used: transforming growth factor beta 1 (TGF-β1, cat# Mm01178820_m1), interleukin-4 (IL-4, cat# Mm00445259_m1), interleukin-10 (IL-10,cat# Mm00439614_m1), interleukin-1β (IL-1β, cat# Mm00434228_m1), interferon γ (IFN-γ, cat# Mm01168134_m1), interleukin-6 (IL-6, cat# Mm00446190_m1), chemokine (C-C motif) ligand 2 (CCL2, cat #Mm00441242_m1), chemokine (C-C motif) ligand 3 (CCL3, cat # Mm00441259_g1), chemokine (C-C motif) ligand 4 (CCL4, cat # Mm00443111_m1), chemokine (C-C motif) ligand 7 (CCL-7, cat # Mm00443113_m1), chemokine (C-C motif) ligand 9 (CCL9, cat # Mm00441260_m1), chemokine (C-C motif) ligand 22 (CCL22, cat # Mm00436439_m1), C-X-C motif chemokine 10 (CXC 10,cat # Mm00445235_m1), C-C chemokine receptor type 2 (CCR2, cat # Mm99999051_gH), chemokine (C-X-C motif) receptor 2 (CXCR2, cat # Mm99999117_s1), arginase 1 (Arg-1, cat # Mm00475988_m1), Chitinase 3-like (CHI3, cat # Mm00657889_mH), and 18S rRNA (cat # Mm03928990_g1). Expression of the target genes was determined by qRT-PCR using Gene-specific TaqMan Assay Reagents and TaqMan Gene Expression Assay products on a 7900 HT Fast Real time PCR system (Applied Biosystems, Foster City, CA, USA). Real time PCR was conducted using a 384-well plate (Micro- Amp Fast Optical 96-well Reaction plates and MicroAmp Optical Adhesive Film, both from Applied Biosystems). Reactions were performed in triplicate. Each 10 μl reaction contained 0.5 μL 20× TaqMan gene expression assay, 5 μl 2× TaqMan universal Master Mix, and 4.5 μl of cDNA template. Following one initial step of 95 °C for 20 s, the cycling parameters were 95 °C for 1 s, 60 °C for 20 s, and 40 cycles; the data were analyzed using Sequence Detection Systems software (Applied Biosystems), and the cycle number at the linear amplification threshold (Ct) of the endogenous control (18S ribosomal RNA, Applied Biosystems) gene and the target gene was recorded. Relative gene expression (the amount of target, normalized to the endogenous control gene) was calculated using the comparative Ct method formula 2^−∆∆Ct^. All PCR data are reported as mean ± SEM relative expression values.

### Western blotting

Lysates were prepared from brain samples or microglia (EOC13.31) cell lines in chilled RIPA buffer (25 mM Tris-HCL pH 7.6, 150 mM NaCl, 1% NP-40, 1% sodium deoxycholate, 0.1% SDS; Thermo Scientific, Rockford, IL) containing protease inhibitor (complete Mini, EDTA-free Protease inhibitor cocktail tablets, Roche Diagnostics, Indianapolis, IN). Samples were homogenized (3 cycles of 5 s each) using a cordless pellet pestle motor (Kontes, Fisher Scientific, Pittsburgh, PA) and allowed to lyse for 30 min on ice, followed by centrifugation at 13.2 (×10,000) rpm for 15 min (Eppendorf Centrifuge 5415 D, Eppendorf North America, Hauppauge, NY), and the cleared supernatant was collected and stored at − 20 °C. Nuclear and cytoplasmic proteins were extracted separately using the NE-PER Nuclear and cytoplasmic Extraction Reagents (Thermo scientific). Protein concentrations were determined using BCA kit (Pierce BCA Protein Assay Kit; Thermo Scientific, Rockford, IL). Fifteen to 20 μg of protein samples were separated on a SDS polyacrylamide gel (10–15%) and subsequently electrophoretically transferred onto nitrocellulose membranes (Thermoscientific), followed by blocking with nonfat dry milk in TBST (50 mM Tris-Hcl, pH 7.4, 150 mM NaCl, 0.2% Tween 20). The membranes were incubated overnight with any of the mentioned primary antibodies: rabbit anti mouse Nrf-2 (1:1000; Thermo Fisher Scientific, cat# PA5-27882), rabbit anti mouse 4HNE (1:1000; Abcam, cat# ab46545), rabbit anti mouse Phospho-NF-κB p105 (1:1000; Cell Signaling, cat#4806), rabbit anti mouse NF-κB p105 p105/p50 (1:1000; Cell Signaling, cat#13586), rabbit mAb NF-κB p65 (1:1000; Cell Signaling, Cat#8242), Phospho-NF-κB p65 (1:1000; Cell Signaling, Cat#3033), and anti-mouse ß-actin(1:10,000; Sigma cat#A5441), followed by HRP labeled goat anti mouse or goat anti rabbit or goat anti sheep (1:1000; Santa Cruz). Protein bands were detected using a chemiluminescence (ECL kit) method (Pierce) and visualized on x-ray film (Kodak).

### Cytokine ELISA

The concentration of the following cytokines in the brain lysates were determined using ELISA kits according to the manufacturer’s instructions: IL-10 (ThermoFisher Scientific, cat# KMC0101), IL-6 (ThermoFisher Scientific, cat# KMC0061), IFN-γ (ThermoFisher Scientific, cat# KMC4021), TNF-α (ThermoFisher Scientific, cat# KMC3011), and IL1β (ThermoFisher Scientific, cat# KMC0011).

### Cell viability

Microglia were seeded at a density of 5000 cells/well in a 96-well plate, and the cell viability was determined using MTS assay (Promega; cat#G3580). Briefly, at the end of treatment regimen, 100 μl of media was removed and 20 μl of Cell Titer 96Aq reagent was added. The plates were incubated for 1.5 h at 37 °C to allow the MTS tetrazolium compound to convert into a colored soluble formazan. Absorbance was recorded at 490 nm using a microplate reader (Spectra Max, Molecular Devices).

### Cellular glutathione content

GSH levels were determined as described by Kamencic et al. with slight modification [33]. Cells were cultured in 96-well plates as described above and subjected to various treatments, after which wells were washed with PBS and incubated with 40 μM monochlorobimane (MCB) in the dark for 30 min at 37 °C, followed by two further washes with PBS. Fluorescence intensity was measured using a spectrofluorophotometer microplate reader (SpectraMax, MS Molecular Devices), with excitation and emission wavelengths of 405 and 510 nm, respectively. Samples were assayed in triplicates.

### Reactive oxygen species (ROS) detection

ROS generation was measured by labeling cells with CellROX Deep Red Reagent following the manufacturer’s instructions (Molecular Probes, Life Technologies). CellROX deep red is a cell permeable non-fluorescent dye that is oxidized by cytoplasmic free radicals to emit fluorescence. Microglia were grown on 35-mm glass bottom dishes (MatTeK Corps). After various treatments, cells were washed twice and loaded with 5 μM of the fluorescent probe for 1 h at 37 °C. Cells were washed three times with PBS and imaged immediately. Multiple random images were captured using an Olympus FV1000 confocal microscope equipped with a HeNe 635 nm laser, 20× objective, NA 0.75 with an electronic zoom of 1.2. Laser intensity, scan speed, and other settings were attenuated to minimize phototoxicity and photobleaching. In addition, for reproducibility and comparison purposes, all microscope settings were kept identical across all treatment groups.

### Analysis of cell morphology

Differential Interference Contrast (DIC) images of microglia grown on 35-mm glass bottom dishes were captured using a Fluoview FV 1000 Olympus confocal microscope equipped with HeNe laser, 20× objective, NA 0.75. Morphological analysis was conducted as described by McWhorter et al. [34]. Briefly, NIH ImageJ software was used to trace and measure long and short axis of each cell manually. The long axis was defined as the longest length of the cell, and the short axis was defined as the length across the nucleus in a direction perpendicular to the long axis. The ratio of the two axes was determined and considered as the elongation factor.

### Immunofluorescence microscopy

After treatment, cells were fixed in 4% paraformaldehyde for 20 min, then permeabilized with 0.2% saponin in 10% FBS-PBS for 20 min at room temperature, and stained with goat anti Arginase 1 (1:200; Santa Cruz, cat# SC18355), goat anti IL-1β (1:300; Abcam, cat #ab195991), and a secondary antibody conjugated with Alexa Fluor 647 or Alexa Fluor 488 (1:200, Abcam). Cells were mounted using VectaShield mounting medium containing DAPI (Fisher Scientific), and images were visualized using an Olympus FV1000 confocal microscope with a 60× PlanApoN objective, NA 1.42 using inbuilt 405-nm diode, 488-nm argon, and 635-nm diode laser settings. Images were captured sequentially with a scan speed of 12.5 μs/pixel. Acquisition settings were offset to minimize photobleaching and also set using appropriate iso-controls. In addition, microscope settings were kept identical for each treatment group.

### Statistics

Statistical analyses were performed using either Stata 11(College Station, TX) or sigma plot 12.0. Student’s T test or one-way analysis of variance or Student-Newman-Keuls Method were applied for pairwise multiple comparisons. All data are presented as mean ± SEM. Compounded.

## Results

### Maternal exposure to a single episode of GSH imbalance increases ethanol-induced oxidative damage in the fetal brain

**Fig. 2:**
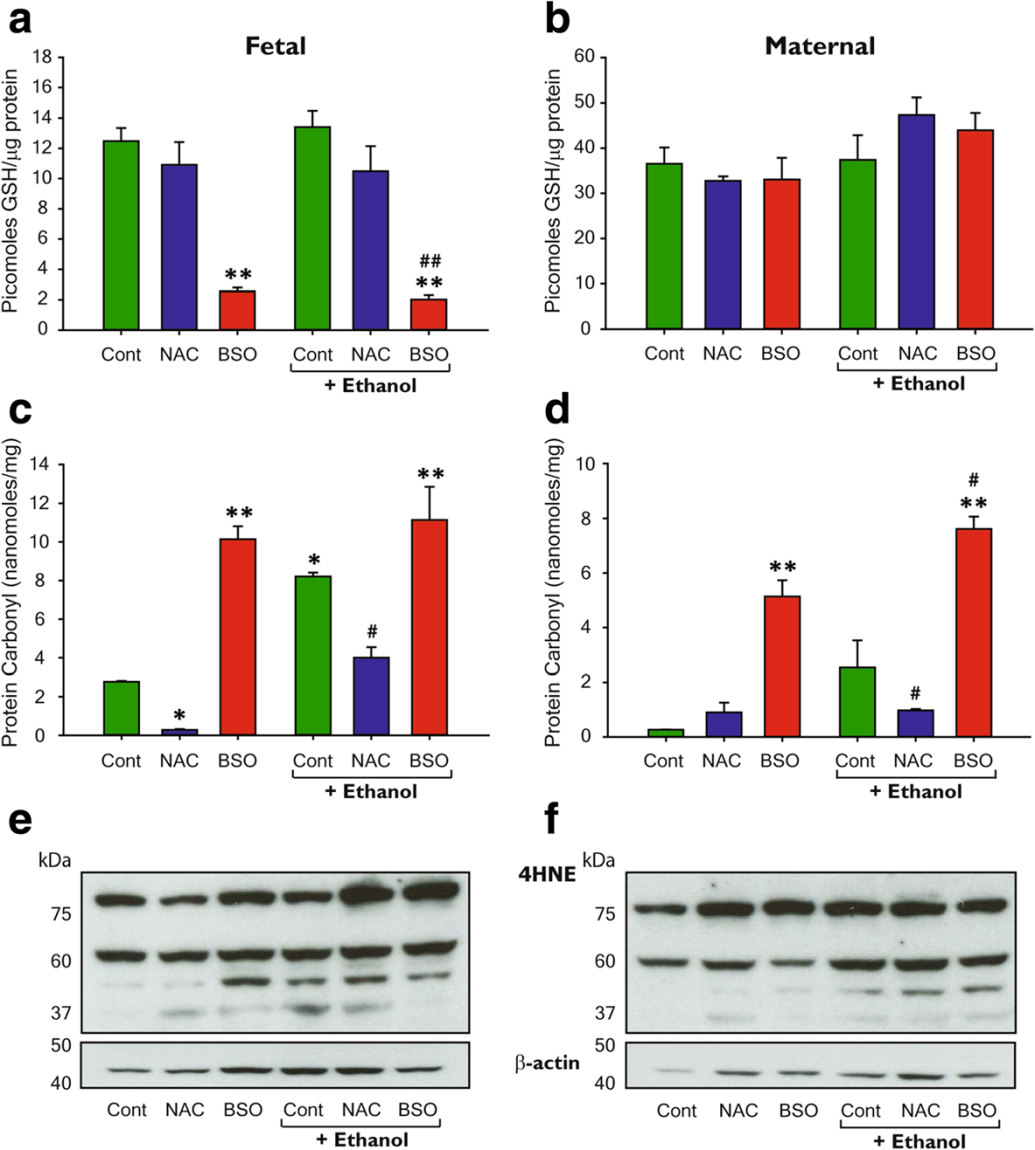
Ethanol and glutathione depletion generate oxidative stress in fetal and maternal brain. Twenty-four hours after dams were subcutaneously administered ethanol (2.5 g/kg), following 1 h intraperitoneal pretreatment with either NAC (4 mg/dose) or BSO (1.5 g/kg), total GSH levels were determined by HPLC in fetal (**a**) and maternal (**b**) brain lysates. 2,4-Dinitrophenylhydrazine (DNP) derivatized protein carbonylation levels in lysates were quantified spectrophotometrically in fetal (**c**) and maternal (**d**) brains. Expression of 4-Hydroxynonenal adduct formation was used to determine lipid peroxidation in the brain. A representative immunoblot against 4HNE in fetal (**e**) and maternal (**f**) lysates. Values are mean ± SEM (*p ≤ 0.05, **p ≤ 0.005 vs control; #p ≤ 0.05, ##*p* ≤ 0.005 vs ethanol), (*n* = 6 per group)

The susceptibility of the fetal brain to oxidative stress produced by ethanol is likely to be augmented by its underdeveloped antioxidant machinery. Therefore, as a first step, the contribution of maternal antioxidant status to oxidative damage in the fetal brain was evaluated by measuring the levels of reduced glutathione and protein and lipid peroxidation products (carbonyls and 4-HNE adducts) at 24 h, following a single episode of moderate ethanol exposure in dams pretreated with two GSH modulators, NAC or BSO. NAC is a weak ROS scavenger that supplies cysteine for GSH synthesis, while BSO is an irreversible inhibitor of γ-glutamylcysteine, the rate-limiting step in GSH synthesis, and is extensively used to deplete GSH levels. Ethanol treatment alone did not produce any significant change in GSH levels in the fetal brain. Compared to control or ethanol-treated group, pretreatment of dams with BSO alone or with ethanol significantly reduced the GSH content by 79.5 and 83.8% in the fetal brain (*p* < 0.005). However, no changes in GSH levels were observed in the maternal brains from either treated or control mice (*p* < 0.08) (Fig. 1b). The presence of protein carbonyls and 4-HNE represents the extent of oxidative damage induced by ROS. The decrease in GSH levels following BSO pretreatment corresponded with increased protein and lipid peroxidation products, like 4-HNE, in fetal brain homogenates. Carbonyl content was sharply increased in the fetal brains exposed to BSO (+ 365%; *p* < 0.005), BSO + EtOH (401%; *p* < 0.005), and EtOH (+ 295%; *p* < 0.05) when compared to control (Fig. 2c). Notably, maternal brain was resistant to protein oxidation when exposed to ethanol alone (*p* < 0.060). However, administration of BSO before EtOH exposure significantly enhanced the levels of protein carbonyls (*p* < 0.005) in comparison to the control or the ethanol-treated group (Fig. 2d). Ethanol-induced protein oxidation was completely prevented by supplementation with NAC in both fetal and maternal brain (Fig. 2c, d). Furthermore, we probed immunoblots of brain homogenates with antibodies directed against 4-HNE, a cytotoxic breakdown product of fatty acid peroxides, and found levels of 4-HNE were noticeably increased in both fetal and maternal brains exposed to EtOH, BSO, or BSO + EtOH, when compared with control (Fig. 2e, f) as evidenced by the presence of protein bands of approximately 76, 60, 50, and 37 kDa. This effect was reversed by pretreatment with NAC similar to carbonyl content in the control group.

### Oxidative-redox shift causes divergent immune responses in fetal and maternal brain

**Fig. 3:**
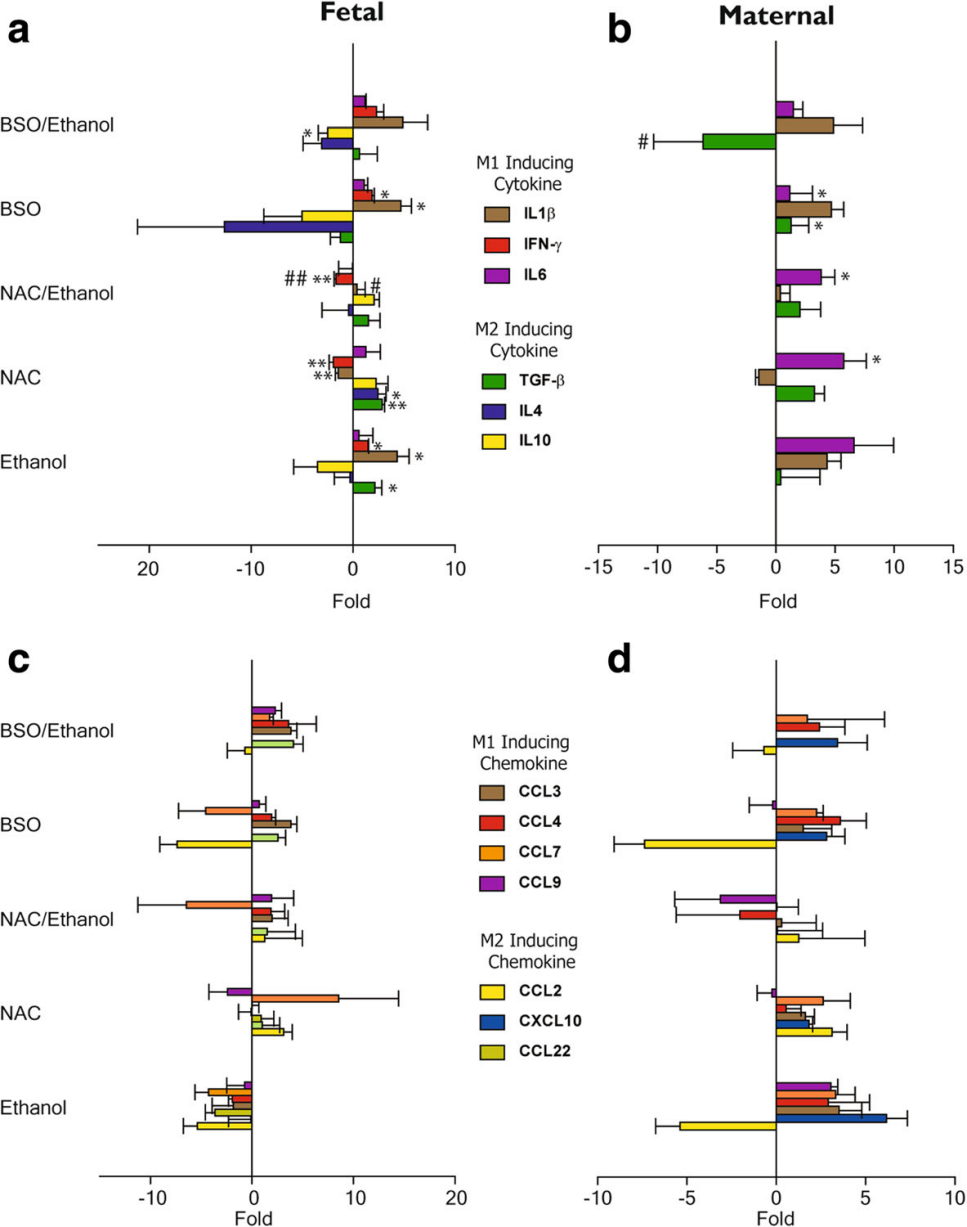
Effect of Ethanol and glutathione modulation on inflammatory response in fetal and maternal brain. Gestational exposure to ethanol and GSH variation has a direct consequence on fetal cytokine/chemokine RNA expression as determined by qRT-PCR. Levels were normalized to 18S rRNA and expressed relative to control. Cytokine and chemokine response in the fetal (**a**, **c**) and maternal (**b**, **d**) brain, respectively, were further outlined based on their distinct association with microglia M1 or M2 phenotype. Values are mean ± SEM. (*p ≤ 0.05, **p ≤ 0.005 vs control; # *p* ≤ 0.05, ## *p* ≤ 0.005 vs ethanol) (*n* = 4–6 per group)

Redox imbalance and oxidative stress are known to elicit transcriptional induction of inflammatory genes [28]. Thus, we initially set to evaluate the effect of ethanol and GSH modulation on selected pro- and anti-inflammatory mRNA expression and also the subsequent changes in cytokine proteins in fetal and maternal brain. To determine changes in gene expression for each treatment regimen, values are expressed as fold change over the corresponding control and analyzed using independent Student’s *t* test. Since differential expression of inflammatory mediators are known to drive microglia to acquire either M1-like or M2-like phenotypes [35], we grouped these markers according to their association with distinctive microglia phenotype M1 (IL-1β, IFN-γ, IL-6, CCL3, CCL4, CCL-7, CCL9,) or M2 phenotype (TGF-β, IL-4, IL-10, CCL2, CCL22, CXCL10, Arg1, Chi1, CCR2, and CXCR2). Exposure to BSO or EtOH alone or their combination upregulated the expression of inflammatory cytokines (IL-1β, IFN-γ, IL-6) in the fetal brain, when compared to control (Fig. 3a), although only IL-1β and IFN-γ expression was significantly affected by BSO and EtOH treatments (*p* < 0.05). In contrast, NAC pretreatment significantly downregulated the expression of both, IL-1β and IFN-γ in the fetal brain, in the absence and presence of ethanol (*p* < 0.05 and *p* < 0.005, respectively). Elevated expression of IL-6 was observed across all the groups, though not statistically significant. The most notable anti-inflammatory M2 cytokine was IL-10: its expression was significantly suppressed in the group treated with BSO + EtOH (*p* < 0.05 vs control) (Fig. 3a). Conversely, the expression of M2-associated cytokines was upregulated in NAC or NAC + EtOH-exposed fetuses. Modulation of these inflammatory markers was less evident in the maternal brain. However, IL-6 was clearly upregulated in NAC, NAC + EtOH, BSO, BSO + EtOH groups, and TGF β was downregulated in BSO + EtOH group (*p* < 0.05). Surprisingly, none of the chemokines measured showed any significant changes in gene expression in either maternal or fetal brain; however, an overall trend of upregulation of M1-associated chemokines (CCL3, CCL4, CCL7, CCL9) and downregulation of M2-associated chemokines (CCL2, CXCL10, and CCL22) was observed in GSH depleted fetal brain. Similarly, NAC pretreatment appeared to inhibit the expression of M1-associated chemokine genes (CCL3, CCL4, CCL9) and simultaneously increase the expression of M2-associated chemokines (CCL2, CXCL10, CCL22) (Fig. 3c).

**Fig. 4:**
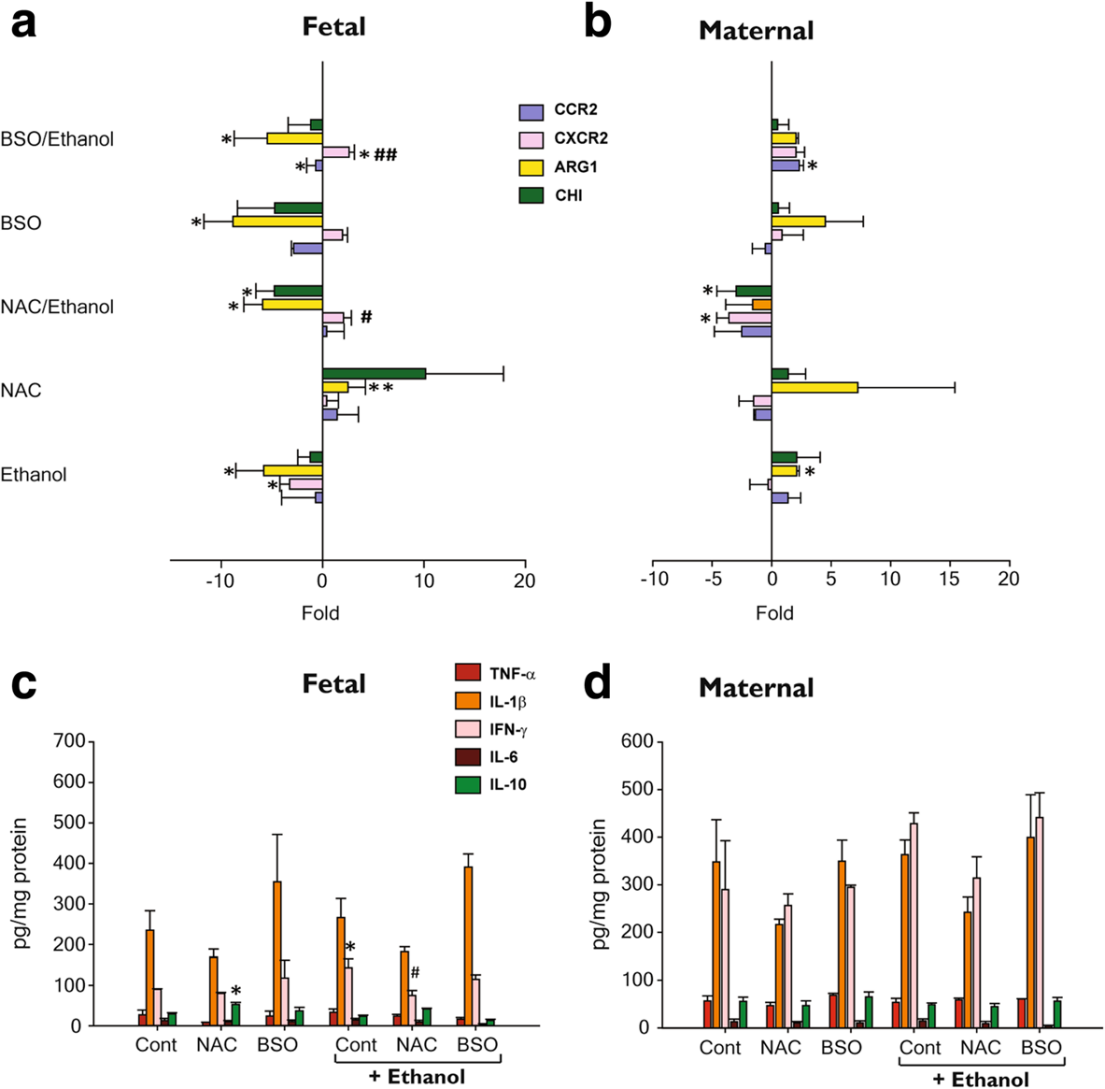
Effect of oxidative stress on inflammatory markers and protein expression. qRT-PCR analysis of M1 (CXCR2) and M2 (Arg-1,Chi,CCR2) specific markers in the fetal (**a**) and maternal (**b**) brain. Expression level of each gene was normalized to 18S rRNA and expressed relative to the control group. Values are ± SEM. Protein level expression of cytokines in the fetal (**c**) and maternal (**d**) brain. Values are mean ± SEM. (*p ≤ 0.05, **p ≤ 0.005 vs control; #*p* ≤ 0.05, ##*p* ≤ 0.005 vs ethanol) (*n* = 6 per group)

We next tested the expression of phenotype-specific markers Arg1, Chi, CCR2, and CXCR2, which indicate the polarization of microglia towards an M2 phenotype. More specifically, Arg1 was significantly induced in fetuses exposed to NAC (*p* < 0.001), when compared to control; however, ethanol treatment mitigates this response. In contrast, Arg1, Chi1, and CCR2 expression were reduced in fetal brain from BSO and BSO + EtOH groups. With respect to maternal brain, the following significant changes were observed in maternal brain: upregulation of Arg1 following EtOH exposure, up-regulation of CCR-2 upon exposure to BSO + EtOH, and suppression of CXCR-2 and Chi with NAC exposure (*p* < 0.05) (Fig. 4a, b).

Cytokines such as IL-1β, TNF-α, IL-6, IL-10, and IFN-γ also play a key role in neuronal differentiation [36], survival [37], and pathological profile that emerges in later life [38]. Therefore, we next determined protein expression of these cytokines by ELISA in lysates of fetal and maternal brain. Ethanol exposure increased IFN-γ (*p* < 0.05), but this effect was mitigated by NAC pretreatment (*p* < 0.025). A M2 phenotypic (IL-10) response was observed in fetal brain from the group with NAC exposed alone (*p* < 0.025). High levels of IL-1β are known to occur in the developing brain, and these levels taper off towards the end of gestation [39]. No significant differences in relative cytokine protein levels were detected in maternal brain lysates between the treatment groups (Fig. 4d).

### Nrf-2 activation promotes cell survival despite loss in GSH and increased OS

**Fig. 5:**
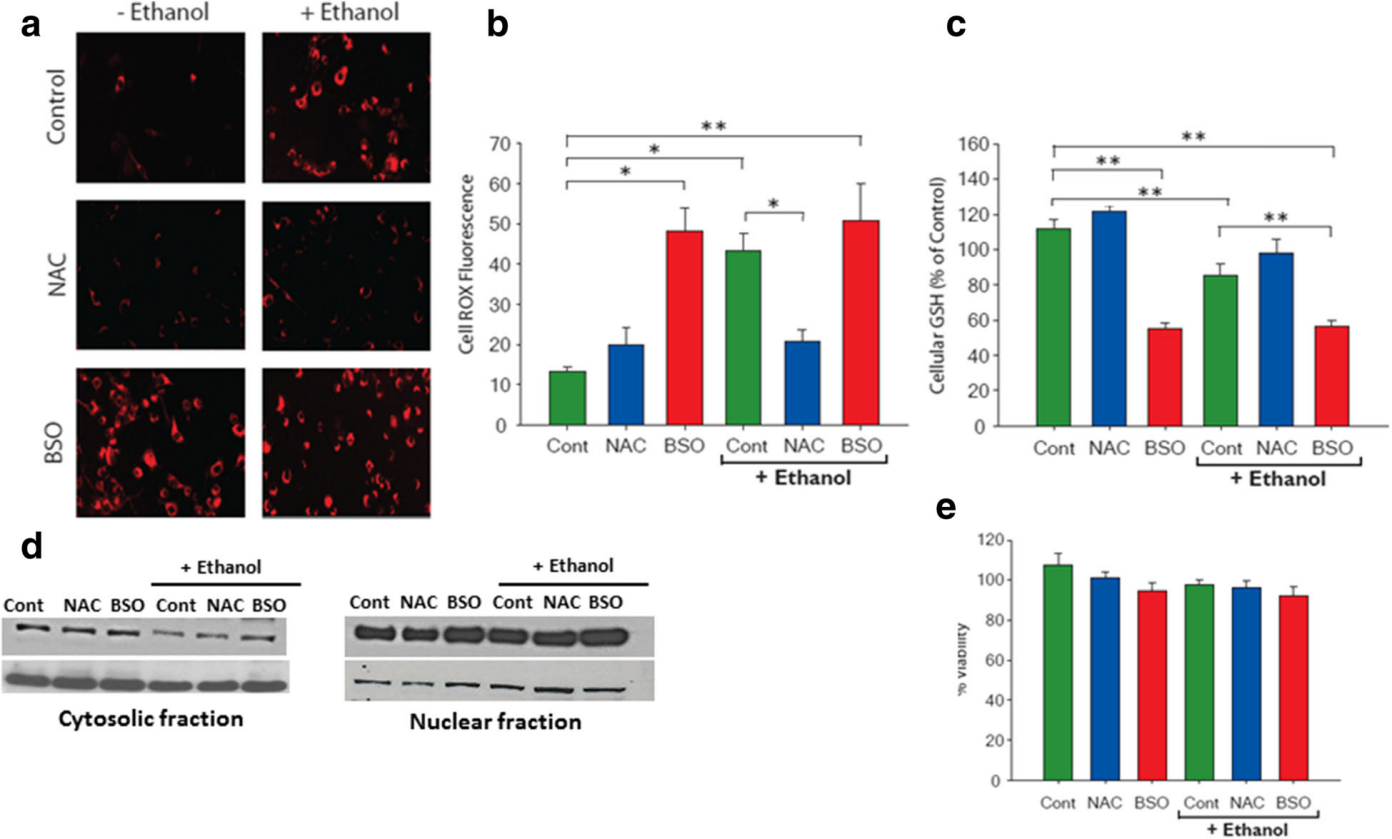
Loss of GSH content in microglia influences oxidative stress but not cell viability. EOC13.31 microglia cells were divided into six groups: either untreated (control) or pretreated with 500 μM NAC or 200 μM BSO for 18 h, prior to 22 mM EtOH exposure for 6 h. Confocal images (20×) of cells labeled with CellROX Deep Red Reagent to detect ROS generation (**a**). Semi-quantification analysis of ROS fluorescence intensity (**b**). Intracellular GSH levels were determined fluorometrically by labeling cells with 50 μM Monochlorobimane (**c**). Western blot analysis for Nrf-2 expression and nuclear translocation, where Lamin B1 and β actin was used as a loading control for nuclear and cytosolic fraction, respectively (**d**). Effect of the above treatment regimen on cell viability (MTS assay) (**e**). Values are presented as mean average ± SEM three independent experiments. Student-Newman-Keuls comparison analysis was used (*p ≤ 0.05; **p ≤ 0.005)

In order to identify key components of the inflammatory cascade in the fetal brain, we next employed an in vitro model using microglia EOC13.31 cells subjected to similar ethanol-induced oxidative stress with varying redox status, in the presence or absence of NAC or BSO pretreatment. Initially, cell toxicity of various concentrations of ethanol was tested using MTS assay which indicated a significant loss in cell viability of microglia at 44 mM ethanol and above (Additional file 1: Figure S1). Therefore, an optimum non-cytotoxic dose of 22 mM ethanol for 6 h was selected for all subsequent experiments. This dose is physiologically relevant in humans and is reflective of blood alcohol levels reported during EtOH intoxication [40]. A comparison of oxidative stress levels in microglia between various groups was determined using confocal microscopy (Fig. 5a). Quantitative analysis of the fluorescence intensities by Image J (Fig. 5b) demonstrated an increased ROS generation in microglia exposed to EtOH and BSO alone (*p* < 0.05) or in combination (*p* < 0.005), as compared to control (saline). Interestingly, the fluorescence signal was diminished in cells treated with NAC+ EtOH, as compared to EtOH group (*p* < 0.05). Since oxidative stress rapidly converts reduced GSH into its oxidized GSSG state, thus depleting the total cellular GSH pool, we also measured GSH levels using the fluorochrome monochlorobimane (Fig. 5c). Exposure to EtOH and BSO either alone or in combination significantly reduced intracellular GSH content (*p* > 0.005). Similar to in vivo conditions, intracellular GSH levels were neither significantly altered after overnight NAC pretreatment alone nor perturbed when NAC treatment was followed by EtOH exposure (Fig. 5c). We next examined the expression of Nrf-2, a transcription factor known to trigger expression of several antioxidant enzymes which is an important cellular defense and survival mechanism against oxidative stress, especially following EtOH and BSO exposure. Western blot analysis in Fig. 5d shows an increase in nuclear accumulation of Nrf-2 expression in microglia pretreated with BSO and/or exposed to EtOH that was concurrent with a decline in cytosolic Nrf-2. Thus, it appears that GSH depletion facilitates the accumulation of Nrf-2 in the nucleus. Consistent with this result, expression of CuZnSOD, an antioxidant, was enhanced in GSH depleted cells exposed to EtOH (Additional file 2: Figure S2), which is indicative of a concurrent defense mechanism to quench the free radicals being generated and thus inhibiting ROS-mediated cytotoxicity (Fig. 5e).

### Microglia GSH content impacts differential expression of M1 and M2 markers and morphological changes

**Fig. 6:**
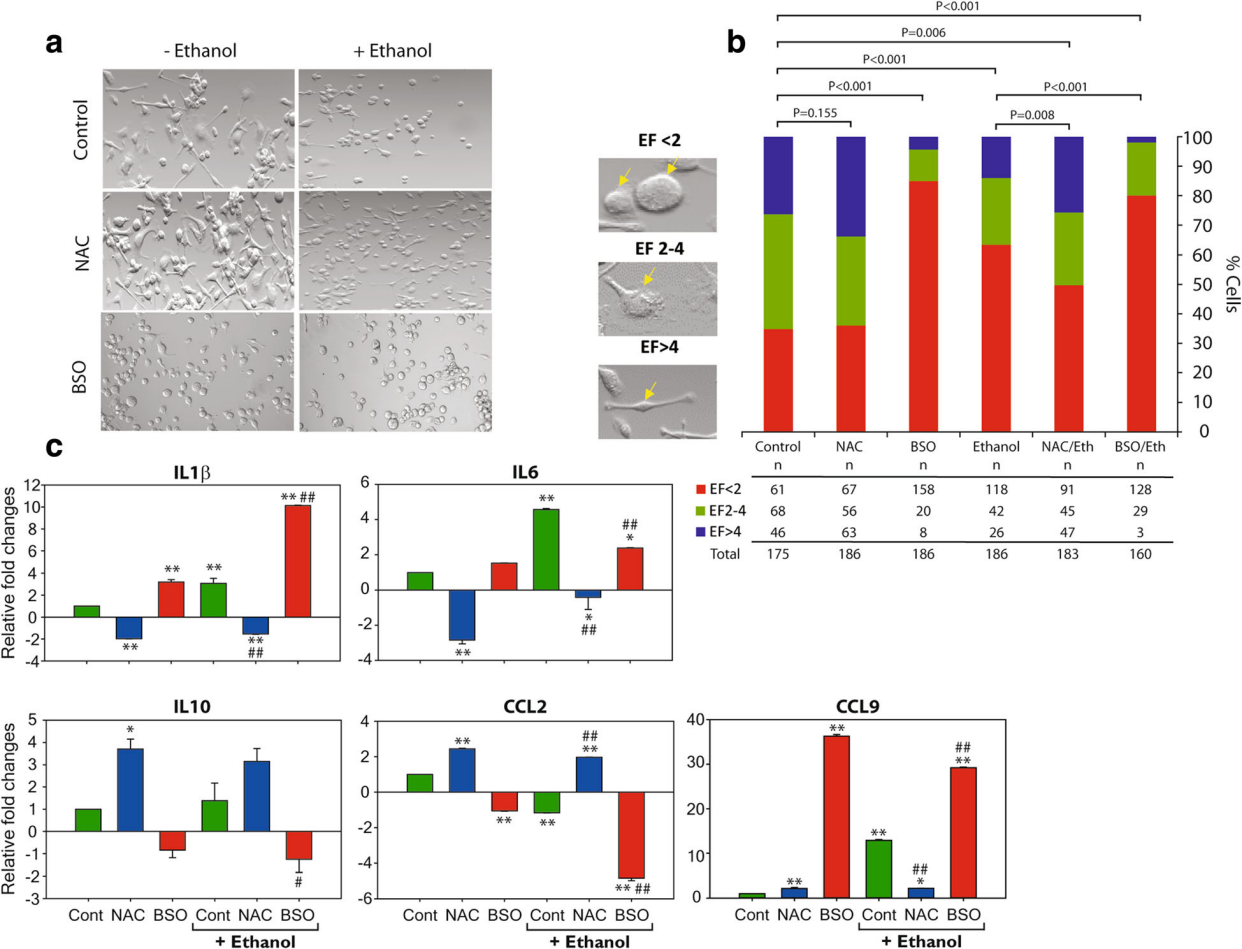
Redox status alters microglia morphological phenotypic changes. Differential interference contrast (DIC) images of EOC13.31 (20×), cells were either untreated (control) or pretreated with 500 μM NAC or 200 μM BSO for 18 h, prior to 22 mM EtOH exposure for 6 h. (**a**). Elongation factor was calculated by measuring the length of the long axis divided by the length of the short axis (**b**). Values obtained were divided into three categories, and a reference image of each category is depicted along the Y-axis. qRT-PCR was conducted of selected M1 (IL-1β, IL-6, CCL9) and M2 (IL-10, CCL2) markers. Expression level of each gene was normalized to 18S rRNA and illustrated relative to the control group (**c**). Values are presented as mean ± SEM of three independent experiments (*p ≤ 0.05; **p ≤ 0.005)

**Fig. 7:**
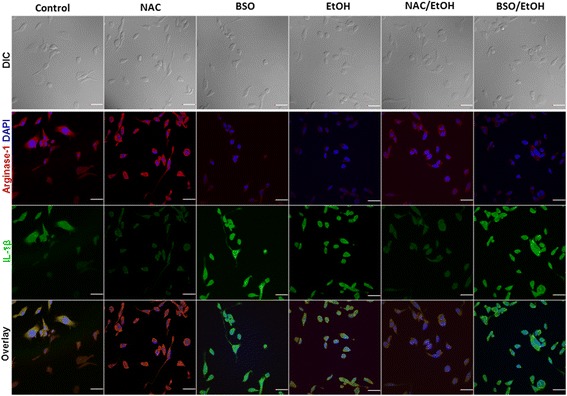
Oxidative stress and intracellular GSH dictate M1/M2 phenotypic acquisition of microglia. EOC 13.31 cells grown on coverslips were pretreated with either NAC (500 μM) or BSO (200 μM) for 18 h, followed by either absence or presence of EtOH (22 mM) exposure for 6 h. Immunohistochemistry was performed using antibodies against IL-1β (M1), Arginase1 (M2) and subsequently visualized with Alexa Fluor 488 (green) and Alexa Fluor 647 (red) labeled secondary antibodies, respectively. DAPI was used as a nuclear stain. Images were captured with a confocal microscope using a 60× objective. Scale bar = 40 μm. Images shown are representative from three independent experiments

Microglia are morphologically dynamic cells whose morphological changes appear to be closely associated with their functional activities. Overall, under normal conditions, microglia grow as a mixed population, consisting predominantly of bipolar rod-like structures, some with extensive ramifications, in addition to a small percentage of rounded cells. Both representative confocal DIC images (Fig. 6a) along with quantification of the morphological observations (Elongation factor (EF)) (Fig. 6b) demonstrate that oxidative stress whether induced by EtOH or BSO or their combination had a prominent influence on microglia morphology *p* < 0.001. While ethanol exposure caused microglia to predominantly assume a round-amoeboid shape, with fewer cells retaining ramifications, BSO exposure caused almost 85% of cells to become rounded. Despite noticeable cellular elongations and extensive ramifications, the presence of NAC did not yield any significant difference (*p* = 0.155), as compared to control (Fig. 6b). Intriguingly, NAC pretreatment prior to EtOH exposure allowed microglia to retain their ramified state, whether compared to control or ethanol group (*p* = 0.006 and 0.008, respectively). Such obvious morphological changes possibly are interlocked with functional activities; thus, we next examined the mRNA expression of M1/M2-like phenotype signatories driving the microglia polarization states (Fig. 6c). EtOH or BSO exposure significantly increased the gene expression of IL-1β and IL-6, both M1 pro-inflammatory markers. Conversely, NAC pretreatment significantly downregulated the expression of IL-1β and IL-6 and, as expected, upregulated IL-10 and CCL2, both markers of M2 polarization. Consistent with this, immunofluorescent microscopy images of microglia exposed to EtOH, BSO alone, or their combination showed IL-1β expression and, concurrently, an extremely low expression of Arg1, an M2 marker (Fig. 7). On the other hand, NAC treatment inhibited the expression of M1 markers (Fig. 7 and Additional file 3: Figure S3) while simultaneously enhancing Arg1 expression. Taken together, these findings demonstrate that ROS and intracellular GSH directly regulate microglia phenotype, expression of inflammatory markers, and the positive relationship between M2 polarization and cell elongation.

### Activation of NF-κB contributes to acquisition of pro-inflammatory phenotypes

**Fig. 8:**
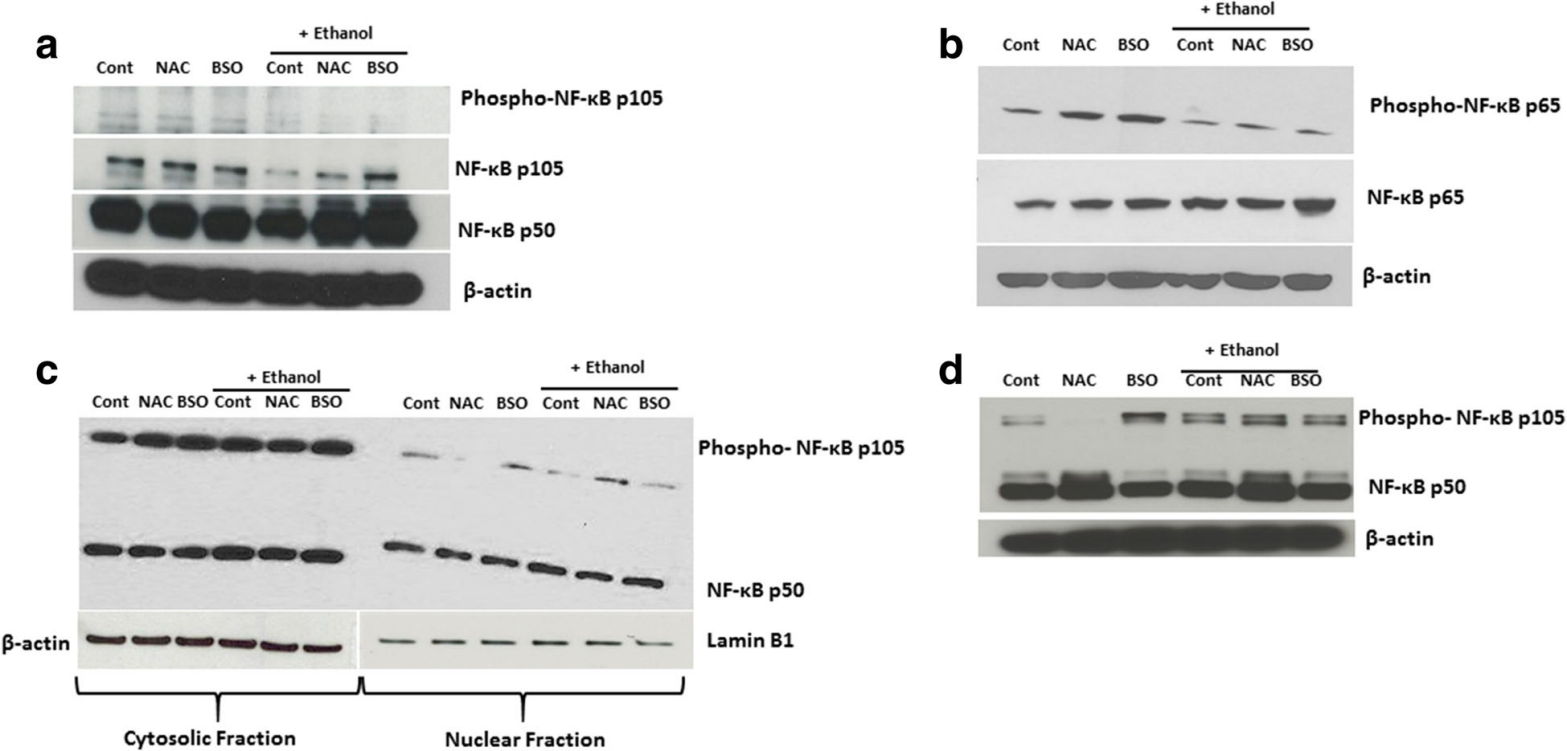
Suppression of GSH triggers NF-κB activation both in in vitro and in vivo. EOC cells were either untreated (control) or pretreated with 500 μM NAC or 200 μM BSO for 18 h, prior to 22 mM EtOH exposure for 6 h. Western blot analysis was performed to determine the expression and activation of NF-κB p105 (**a**) phosphorylated p65 at ser^536^ and total p65 protein (**b**). β-actin served as a loading control. In similar experiments, nucleus and cytosol fractions were separated to determine expression levels of p50 (**c**). Lamin B and β-actin served as nuclear and cytosolic loading control, respectively. Representative immunoblot of p105 in fetal brain samples taken 24 h after dams were pretreated 1 h with either NAC (4 mg/dose) or BSO (1.5 g/kg) followed by ethanol exposure (2.5 g/kg, s.c) (**d**)

Depending on the stimuli and cell type, NF-κB signaling serves a dual function in the brain and is directly associated with inflammation, neuroprotection, neurotransmission, cell death, and cell survival [41–43]. The NF-κB family of transcriptional factors are redox sensitive, dysregulated redox homeostasis may impact NF-κB activation and subsequent release of pro-inflammatory mediators by microglia. Thus, in order to understand the underlying mechanism associated with our treatment regimens, we explored the activation and expression of NF-κB subunits: p105, p50, and p65/RelA. Western blotting results show increased processing of p105 into p50 subunit, elevated expression of p65, and nuclear translocation of p50 in BSO + EtOH-treated cells (Fig. 8a–c), suggesting activation of the classical NF-κB pathway in vitro in microglia depleted of GSH which is augmented by exposure to ethanol. Moreover, ethanol treatment dampened the phosphorylation of p105 and p65 across all groups. Consistent with this, we also observed upregulated expression of p50 in fetal brain exposed to EtOH or BSO alone or in combination (Fig. 8d).

## Discussion

Our results reaffirm that oxidative stress [13, 44] is a causal factor for several immunological and neurological impairments [45] observed in FASD. More importantly, these results show that maternal antioxidant status has direct consequences on fetal brain development; therefore, even a minor short-term shift in redox-oxidative balance during gestation increases the possibility of fetotoxicity from ethanol exposure. To this end, moderate single doses of ethanol, NAC, and BSO were administered at gestational days 16–17 in mice. Twenty-four hours later, we determined the extent of oxidative stress and immune status in the brains of dams and fetuses using microglia M1/M2 paradigm comparison. We show that NAC, BSO, and ethanol all had a predominant effect on the fetal brain and less on the maternal brain. Pronounced oxidative damage characterized the fetal brain response to redox imbalance alone and sometimes in conjunction with ethanol exposure, as seen by increased oxidation of both protein and lipids, as well as GSH depletion (Fig. 2). Although maternal ethanol consumption is reported to reduce GSH content of the fetal brain [46, 47], we observed no such effect. Surprisingly, NAC pretreatment used in this study did not alter GSH levels, unlike restoration of GSH levels by NAC as reported by others [48]. These variations in findings are likely due to difference in administration routes and exposure regimens adopted by us and others.

Our work and evidence from earlier studies show that redox imbalance and oxidative stress are central to induction of inflammatory genes [28]. Moreover, when these factors are combined with ethanol, they appear to leave a persisting inflammatory signature in the offspring [15, 49]. A number of earlier studies suggest microglia activation as the source of inflammation and tissue damage following ethanol intake. However, microglial “activation” which is highly dependent on cytokines and chemokines present in the surrounding milieu may or may not necessarily lead to an inflammatory response [12, 23, 24, 50–52]. Therefore, it is important to distinguish that once activated, microglia can acquire either a detrimental neurotoxic (M1)-like phenotype [11, 49, 53, 54] or a neuroprotective (M2)-like phenotype [25, 54]. Low tissue levels of GSH quite often exacerbated by environmental stressors (ethanol exposure) cause microglia activation to trigger the pro-inflammatory response whereas maintaining optimal tissue GSH concentrations not only abate the neurotoxic response of ethanol but also provide cues for switching to an anti-inflammatory response (Figs. 3, 4, and 5). We emphasize again that GSH levels in the maternal brain remain unaltered across all treatment groups; interestingly however, ethanol, BSO, and NAC individually or in combination do elicit M1/M2 immune responses. This is always more striking in the fetal brain when compared to maternal brain (Figs. 3, 4, and 5). Inadequately developed fetal antioxidant system in early to mid-gestational stages is highly vulnerable to oxidative stress [55] and also explains the differential response seen between the maternal and the fetal brain.

Most distinctly, in the present study, we show a complex co-existence and alterations in microglia phenotype, which is primarily driven by GSH status. Notably, oxidative stress enhances the expression of pro-inflammatory cytokines IL-1 β, IFN-γ, and iNOS and simultaneously reduces anti-inflammatory cytokines IL-4 and IL-10, especially in groups exposed to BSO or ETOH alone or in combination (Figs. 3, 4, 6, and 7). These pro-inflammatory cytokines can directly or indirectly cause neuronal death, dysfunction, attenuate neurogenesis (our unpublished data), and impair spatial learning and memory function [21, 26, 27]. Conversely, treatment with NAC triggers the expression of M2-associated markers such as IL-10, IL-4, TGF-β, CCL22, and Arg and reduced the expression of M1-associated signals (IL-1β, IFN-γ, and CCL9) (Figs. 3, 4, 6, and 7). Previous studies with adult rats found no changes in cytokine expression following ethanol exposure [51, 52] concur with our observations, wherein maternal brain tissues showed either a total absence of IL-10, and IFN-γ mRNA levels or an insignificant difference of protein levels (IL-10,TNF-α, IL1β,IFN-γ). Thus, a fully developed maternal antioxidant system is likely to resist transient fluctuations in oxidative stress and inflammation.

To determine the underlying mechanism of microglia activation and its phenotypic changes, we undertook in vitro experiments (Figs. 5, 6, 7, and 8). Exposure to ethanol and GSH depletion, alone or in combination, alters both the morphological and the functional characteristics of microglia cells by inducing ROS production (Figs. 5 and 6). Similar to our in vivo conditions, pretreatment with NAC maintained cellular GSH and, however, mitigated ethanol-induced ROS generation. In addition, ethanol and BSO exposure generate ROS (Fig. 5a, b) and deplete GSH levels (Fig. 5c). Ethanol augments ROS generation through the activation of NADPH oxidase (data not shown) [20]. GSH serves as a redox buffer against ROS [56], and its depletion is a distinctive feature of apoptotic cell death [57]. However, in this study, BSO treatment resulted in depleting cellular GSH pool and magnifying ROS production in microglia without affecting mitochondrial respiration rate and cell viability. This is likely driven in part by activation of Nrf-2 pathways (Fig. 5d) as an adaptive cellular mechanism to counteract GSH perturbation by eliciting the expression of antioxidant genes, including Cu/Zn SOD for ROS removal. A similar regulatory response to GSH depletion has been observed in other cellular models [58, 59]. In addition to aggravating oxidative damage, ROS also acts as a secondary messenger, modifying gene expression critical for survival [60].

Soluble factors in the microenvironment are known to regulate microglia polarization [28]. We found ROS and intracellular GSH to be critical for regulating cellular morphologies and a strong association exists between cell shape and inflammatory status of these cells (Figs. 6 and 7). Confocal data shows cells to predominantly assume round amoeboid shape under conditions of oxidative stress and redox imbalance. Conversely, microglia exhibit bipolar elongation when cellular redox homeostasis is maintained. Importantly, recent studies report the presence of similar bipolar/rod-shaped microglia at the site of injury, during the early phases of brain damage [61–63]. Such morphologies exert neuroprotective effects by producing greater amount of anti-inflammatory cytokines than pro-inflammatory cytokines [63]. Additionally, changes in morphology are closely associated with the functional activity of microglia [64, 65]. Our RT-PCR data shows that microglia with optimum GSH levels express significantly increased levels of IL-10 and CCL2 and subsequently downregulation of inflammatory cytokines and chemokines (IL-1β, IL-6, and CCL9). On the other hand, depletion of cellular GSH results in microglia expressing higher levels of pro-inflammatory factors compared to control cells. Although ethanol induces microglia activation [20, 66], its polarizing potential on microglia has yet not been fully addressed. Our results establish a strong correlation between morphological alterations in microglia with gene expression profile distinctive to either M1- or M2-like phenotypes. The most well-characterized prototypic M2 marker is the expression of arginase1, and that of M1-like phenotype are IL-1β and iNOS [67–69]. Depletion of GSH resulted in a higher expression of M1-associated markers (IL-1β and iNOS) and exposure to NAC promoted Arg1 expression (Fig. 7 and Additional file 2: Figure S2). Together, these data demonstrate that intracellular GSH status influences cell morphology and modulates microglia polarization. Cell shape is reported to influence polarization in macrophages [34]. In agreement with this study, our results show that ethanol-induced morphological changes in microglia are highly attuned with cytokine and intensify its overall effect on M1 polarization state.

A mechanism that emerges from this study is that the plasticity and functional polarization of microglia is strongly influenced by oxidative stress [28], redox status, and pro- or anti-inflammatory factors [70]. Animals with perturbed redox homeostasis are prone to generate higher levels of ROS and acquire M1 phenotype. Acting as secondary messengers, ROS activate NF-κB signaling leading to transcription of pro-inflammatory cytokine genes. The present study revealed “non-classical” pathway for NF-κB activation. Western blot analysis of NF-κB p105/50, phospho NF-κB p105/50, NF-κB p65/RelA, and phospho NF-κB p65/Rel A reveals increased processing of p105 precursor protein via phosphorylation leading to overexpression of p50 and induction and phosphorylation of p65 in GSH depleted group which was further augmented by ethanol exposure. Of the various homo- and heterodimers of NF-κB, the p65/p50 dimer is the best-characterized inducer of pro-inflammatory genes and is fully functional in microglia [28]. On the other hand, p50 and p52 homodimers function as repressors due to their lack of a transcription activation domain [71]. Earlier studies have established p65/p50 as the primary mediator of NF-κB transcriptional activity for pro-inflammatory genes.

**Fig. 9:**
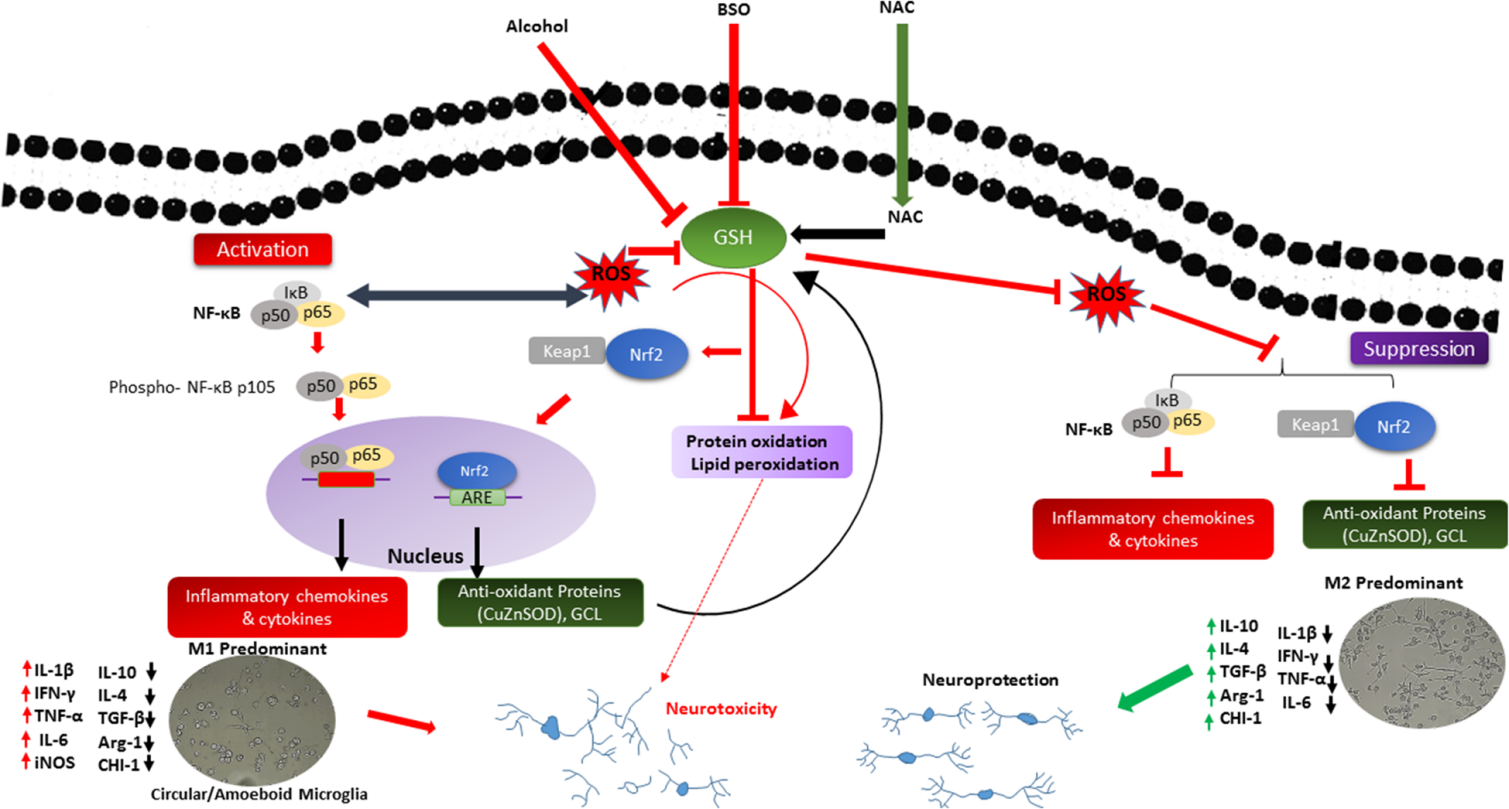
Schematic diagram shows the effect of ethanol, antioxidant disruption on microglia phenotype, and the underlying mechanism in the developing fetal brain. Maternal ethanol consumption acts as an oxidant in the fetal brain by activating the generation of ROS. Also, suboptimal conditions of cellular GSH further aggravates ethanol-induced oxidative stress, ROS accumulation, and Nrf-2 activation. ROS in turn triggers redox-sensitive NF-κB activation by phosphorylation of NF-κB p65 on its two serine residues. Activated NF-κB then induces the production of inflammatory cytokines (IL-1β, IL-6, TNF-α, IFN-γ), chemokines (CCL3, CCL4, CCL7, CCL9) in the fetal brain. Inflammatory milieu drives the microglia cells to a classically activated (M1) phenotype which displays spherical morphology in vivo. Enhanced expression of these inflammatory cytokines, chemokines, iNOS and free radical are key to cytotoxicity and tissue injury. In contrast, optimal or enhanced GSH content in the fetal brain inhibits the damaging mechanism that accompanies maternal ethanol consumption. In addition, it predominantly drives M2 phenotypic changes which exhibit an elongated bipolar morphology with robust expression of (ARG-1, IL-10, TGF-β), the absence (or very low levels) of pro-inflammatory cytokines, and ROS response generally associated with tissue injury. Therefore, poor nutritional status or antioxidant reserves pertaining to maternal-fetal environment amplifies effects of environmental stressors such as ethanol

Expression of pro inflammatory cytokines and chemokines (IL-1β, TNF-α, IFN-γ, CCL9, CCL4, CCL3) subsequently incorporate microglia into the network of detrimental M1 phenotype. When abundant cellular GSH pools exist, M2-inducing signals such as IL-10 generally inhibit the expression of M1 chemokines. On the other hand, IL-10 inhibitory effects rely on both the inhibition of NF-κB [72] and STAT-dependent mechanism [73]. The schematic (Fig. 9) depicts the underlying mechanism of phenotypic acquisition of microglia under perturbed redox status and oxidative stress. We speculate that GSH content and ROS levels in microglia constitute a potential link for the crosstalk between Nrf-2 and NF-κB pathways, the two known crucial factor in regulating microglia dynamic and neuroinflammation [74], thereby driving the immunological phenotypic profiles.

## Conclusion

In conclusion, our study shows that even a slight imbalance in the oxidative-redox homeostasis in the immature fetal brain triggers activation of microglia towards a pro-inflammatory M1 phenotype that persists up to 24 h. On the other hand, maintaining GSH levels triggers anti-inflammatory M2-phenotypic response. Therefore, the existence of a bidirectional feedback loop between Nrf2 activation and phosphorylation of NFkB components p105 and p65, and their nuclear translocation associated with expression of inflammatory markers such as IL1-βand IFN-γ, merits further exploration. Further, time-based studies will facilitate our understanding of the extent of fetal brain damage and whether these effects are reversible.

## Additional files

Additional file 1: Figure S1. Concentration-dependent effect of ethanol on cell viability. Plot of cell viability (MTS assay) obtained following 6 h treatment of cells with various concentration of ethanol. Data represent the average and standard deviation values from three replicate experiments. Where *p ≤ 0.05. (TIFF 92 kb)

Additional file 2: Figure S2. Altered glutathione homeostasis impacts ethanol-induces superoxide dismutase expression in microglia. A representative Western blot of CuZnSOD expression in control and treatment groups. Actin served as the loading control. (TIFF 121 kb)

Additional file 3: Figure S3. Intracellular GSH is pivotal in EtOH-induced phenotypic acquisition of microglia. EOC 13.31 cells grown on coverslips were treated with NAC (500 μM) or BSO (200 μM) for 18 h prior to the presence or absence of EtOH (22 mM) exposure for 6 h. Cells were labeled by antibodies against iNOS and Alexa Fluor 488 labeled secondary antibodies. The nuclei of the cells were counterstained with DAPI. GSH depletion by BSO and EtOH exposure synergistically exaggerate the expression of M1 marker (green) iNOS. Images were acquired on FV1000 confocal microscope equipped with a HeNe laser, 60× objective, NA 1.42 with an electronic zoom of 2. *Scale* bar = 40 μm (TIFF 301 kb)

## Abbreviations

Arg1: Arginase1
BSO: L-Buthionine sulfoximine
CCL2: Chemokine (C-C motif) ligand 2
CCL22: Chemokine (C-C motif) ligand 22
CCL3: Chemokine (C-C motif) ligand 3
CCL4: Chemokine (C-C motif) ligand 4
CCL-7: Chemokine (C-C motif) ligand 7
CCL9: Chemokine (C-C motif) ligand 9
CCR2: C-C chemokine receptor type 2
Chi1: Chitinase1
CUZnSOD: Superoxide dismutase
CXCL10: C-X-C motif chemokine 10
CXCR2: C-X-C chemokine receptor type 2
ELISA: Enzyme-linked immunosorbent assay
FASD: Fetal Alcohol Spectrum Disorder
GSH: Glutathione
HPLC: High-performance liquid chromatography
IFN γ: Interferon gamma
IL-10: Interleukin 10
IL-1β: Interleukin 1 beta
IL-4: Interleukin 4
IL-6: Interleukin 6
NAC: *N*-Acetylcysteine
NFκB: Nuclear factor kappa-light-chain-enhancer of activated B cells
Nrf-2: Erythroid 2-related factor 2
OS: Oxidative stress
ROS: Reactive oxygen species
TGF-β: Transforming growth factor beta 1
TLR: Toll like receptors
