# Supplementary figures and images for: Acute maternal oxidant exposure causes susceptibility of the fetal brain to inflammation and oxidative stress
Source: J Neuroinflammation. 2017 Sep 30;14:195. doi: 10.1186/s12974-017-0965-8 (PMC5622443; doi:10.1186/s12974-017-0965-8)

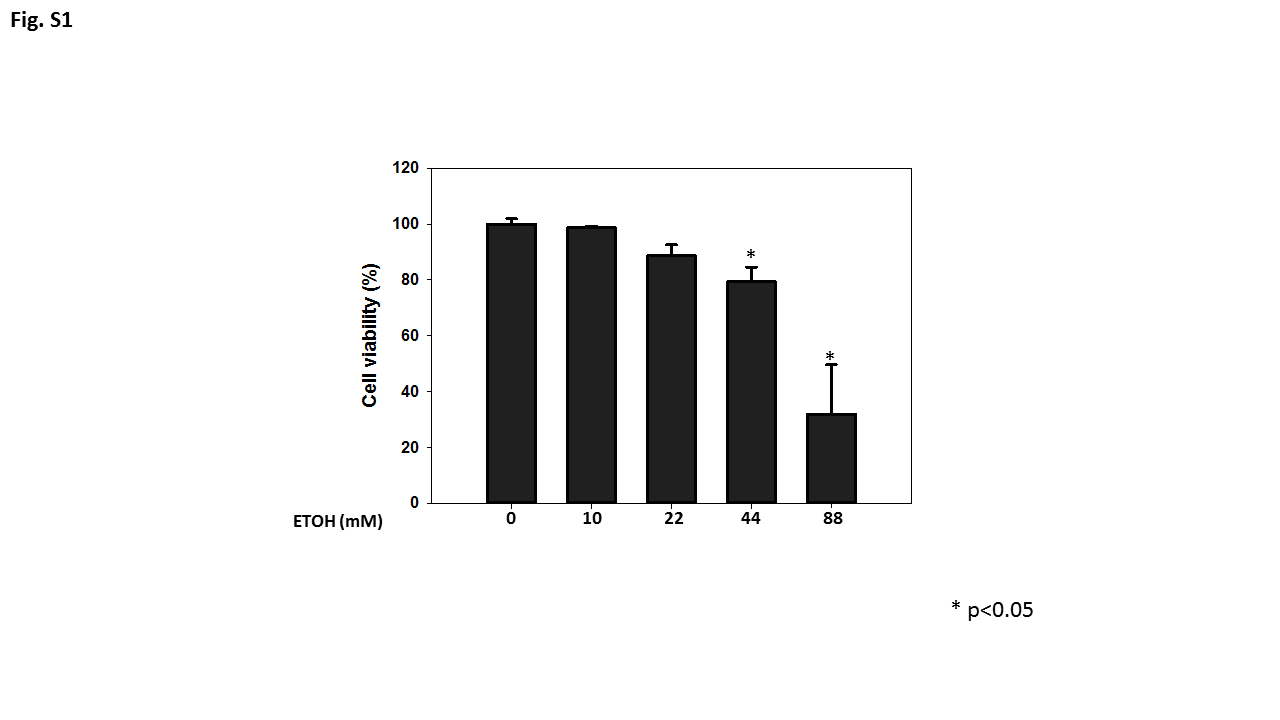

Supplement: Supplementary file 1 — Concentration-dependent effect of ethanol on cell viability. Plot of cell viability (MTS assay) obtained following 6 h treatment of cells with various concentration of ethanol. Data represent the average and standard deviation values from three replicate experiments. Where *p ≤ 0.05. (TIFF 92 kb) [file 12974_2017_965_MOESM1_ESM.tif]

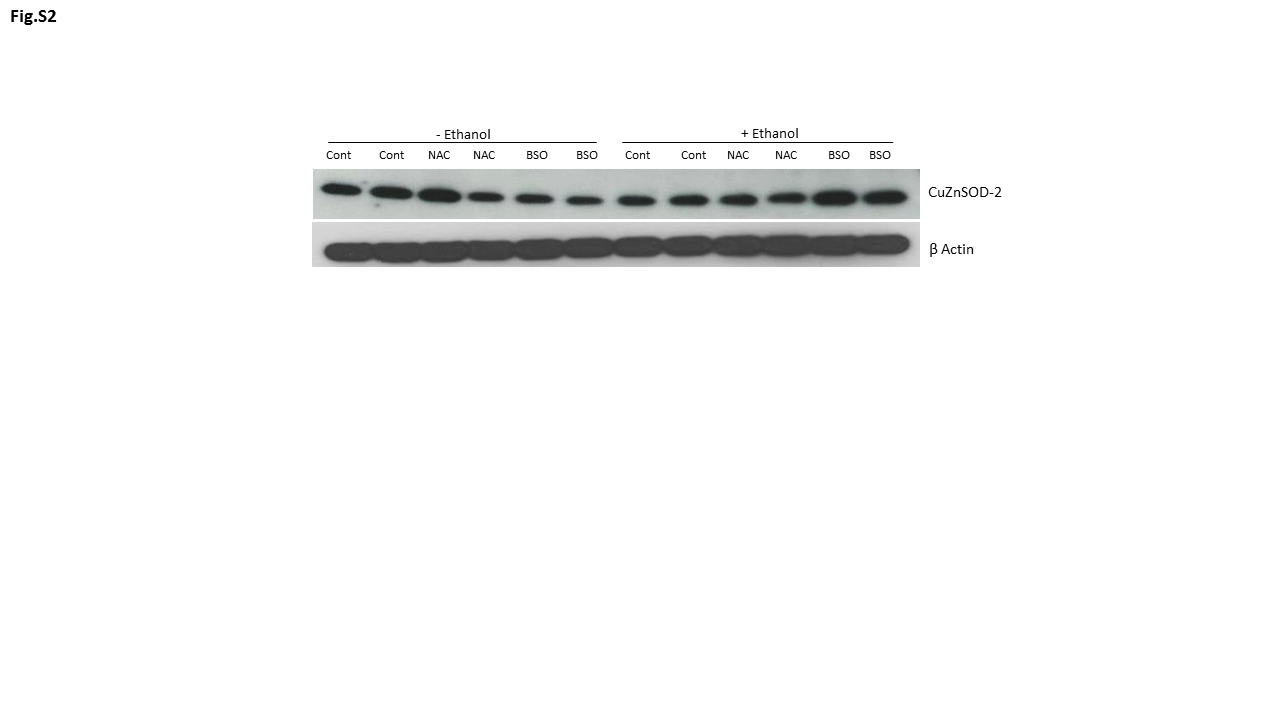

Supplement: Supplementary file 2 — Altered glutathione homeostasis impacts ethanol-induces superoxide dismutase expression in microglia. A representative Western blot of CuZnSOD expression in control and treatment groups. Actin served as the loading control. (TIFF 121 kb) [file 12974_2017_965_MOESM2_ESM.tif]

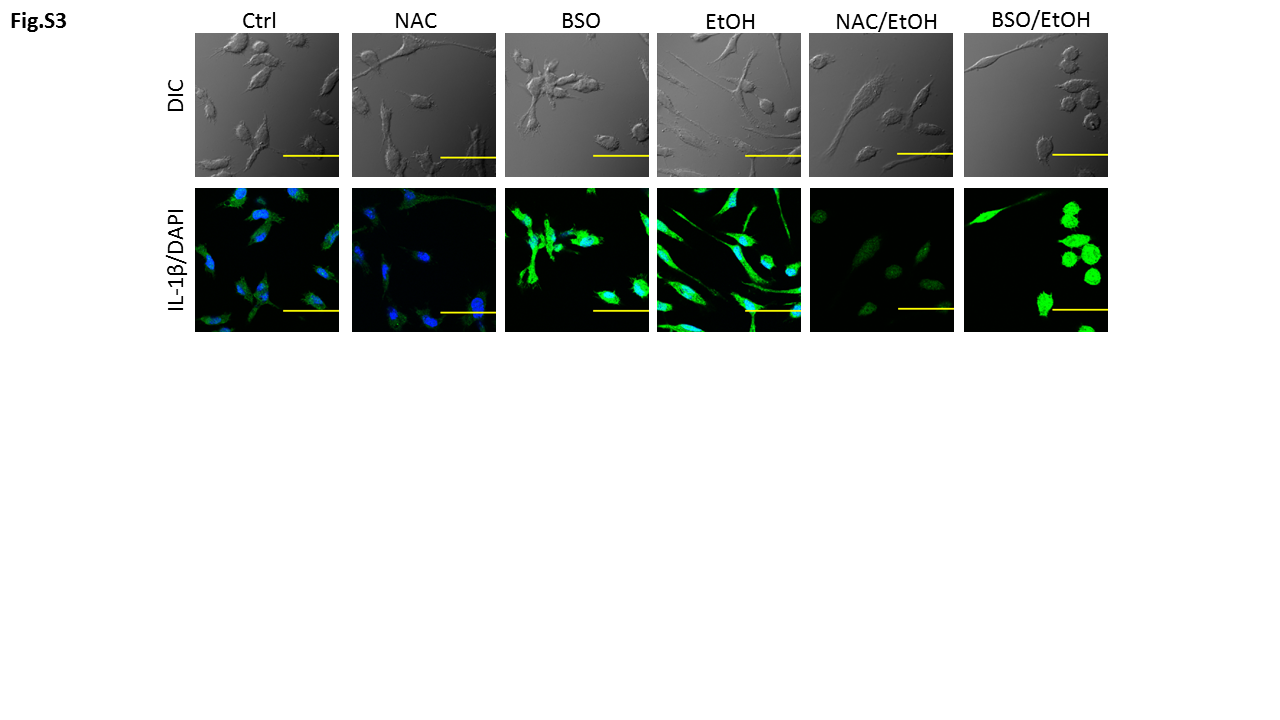

Supplement: Supplementary file 3 — Intracellular GSH is pivotal in EtOH-induced phenotypic acquisition of microglia. EOC 13.31 cells grown on coverslips were treated with NAC (500 μM) or BSO (200 μM) for 18 h prior to the presence or absence of EtOH (22 mM) exposure for 6 h. Cells were labeled by antibodies against iNOS and Alexa Fluor 488 labeled secondary antibodies. The nuclei of the cells were counterstained with DAPI. GSH depletion by BSO and EtOH exposure synergistically exaggerate the expression of M1 marker (green) iNOS. Images were acquired on FV1000 confocal microscope equipped with a HeNe laser, 60× objective, NA 1.42 with an electronic zoom of 2. Scale bar = 40 μm (TIFF 301 kb) [file 12974_2017_965_MOESM3_ESM.tif]
